# Somatic mutations of activating signalling, transcription factor, and tumour suppressor are a precondition for leukaemia transformation in myelodysplastic syndromes

**DOI:** 10.1111/jcmm.17613

**Published:** 2022-11-15

**Authors:** Feng Xu, Lin‐Yun Wu, Juan Guo, Qi He, Zheng Zhang, Xiao Li

**Affiliations:** ^1^ Department of Hematology Shanghai Jiao Tong University Affiliated Sixth People's Hospital Shanghai China

**Keywords:** AML transformation, myelodysplastic syndromes, sequencing, somatic mutations

## Abstract

The transformation biology of secondary acute myeloid leukaemia (AML) from myelodysplastic syndromes (MDSs) is still not fully understood. We performed paired self‐controlled sequencing, including targeted, whole exome, and single‐cell RNA sequencing, in a cohort of MDS patients to search for AML transformation‐related mutations (TRMs). Thirty‐nine target genes from paired samples from 72 patients with MDS who had undergone AML transformation were analysed. The targeted sequencing results showed that 64 of 72 (88.9%) patients presented TRMs involving signalling pathway activation, transcription factors, or tumour suppressors. Of the 64 patients, most of the TRMs (62.5%, 40 cases) emerged at the leukaemia transformation point. Paired whole exome sequencing showed some presumptive TRMs, which were not included in the reference targets in three patients. No patient developed AML only by acquiring mutations involved in epigenetic modulation or ribonucleic acid splicing. Single‐cell sequencing indicated that the activating cell signalling route was related to TRMs in one paired sample. Targeted sequencing defined TRMs were limited to a small set of seven genes (in the order: *NRAS/KRAS*, *CEBPA*, *TP53*, *FLT3*, *CBL*, *PTPN11*, and *RUNX1*, accounting for nearly 90.0% of the TRMs). In conclusion, somatic mutations involved in signalling, transcription factors, or tumour suppressors appeared to be a precondition for AML transformation from MDS.

## 1 INTRODUCTION

In most cases, de novo acute myeloid leukaemia (AML) shows rapid onset without an obvious pre‐AML period.^1^ Patients have been reported to show special genetic abnormalities in different subsets.^2^, ^3^, ^4^ Somatic mutations involving *CEBPa*, *FLT3*, *NPM1*, and *c‐Kit* have been used to assess prognosis.^5^ Based on these tumour‐derived biological characteristics, target‐specific and immunological methods have been developed to treat AML.^5^ In contrast, another group of AMLs showed very different clinical and genetic features. Myelodysplastic syndromes (MDSs) present a haematopoietic status between normal individuals and patients with leukaemia. Indeed, one‐third of people with MDS transform to secondary AML (sAML) when the blasts in their bone marrow are over 20%. Unlike de novo AML, sAML shows unique genetic features.^6^ First, there is often an obvious pre‐AML stage, where somatic gene mutations result in initial events (clonal haematopoiesis) and/or driver events (development of MDS phenotypes), largely involving epigenetic regulation and ribonucleic acid (RNA) splicing.^7^, ^8^ Second, despite preliminary findings on the role of late‐stage gene mutations involving various signalling pathways or transcription during sAML development,^9^, ^10^ including our primary consideration of sAML‐related mutations,^11^ the transformation biology of sAML is still not fully understood.

In a recent review, Menssen et al. analysed previously published mutational profiles of paired MDS and sAML samples, and provided some evidence on transcription factors, and signalling pathway activation genes as potential sAML‐related mutations.^12^ However, there are still many unanswered questions in sAML transformation biology. Could early or late somatic mutations solely involved in epigenetic regulation or RNA splicing induce sAML? Which mutations are the key factors that induce the transformation process? When do transformation‐related mutations (TRMs) emerge? Do they pre‐exist at MDS diagnosis or emerge when the transformation starts? Answering these questions will help us find new insights into sAML pathogenesis and explore some new target therapy strategies. Logically, only systematized findings, but not some sporadic reports, can answer these questions. A systematized finding in this assay means the data should come from a big sample, which could help to focus our attention on some high‐occurrence rate mutations related to sAML transformations. In addition, paired self‐controlled data is required to inform us what mutations initiate MDS development and what other mutations trigger sAML transformation. Finally, this study is focused on the mutated genes related to sAML transformation rather than an overall MDS clonal evolution.^9^ In general, the functions of the mutations that occur during the early/middle stage of MDS (such as those MDS special gene mutations involving epigenetic regulation and RNA splicing) are to initiate clonal haematopoiesis and keep the phenotypes of MDS. In theory, only the late events referring to signalling, transcription, or tumour suppression could start the sAML transformation process.^7^ Therefore, this study aimed to observe what happens when MDS develops into sAML in terms of gene mutations.

Paired self‐controlled samples were acquired at MDS diagnosis and immediately after AML transformation. Sequencing for 39 target genes in all samples was followed by whole exome sequencing in several patients whose targeted sequencing did not identify novel mutations. An additional sample paired was subjected to single‐cell RNA transcription sequencing. Using these techniques, we obtained some useful data regarding the biology of sAML transformation and suggest novel strategies to block the leukaemic transformation of MDS.

## 2 MATERIALS AND METHODS

### 2.1 Sample collection

From January 2004 until October 2020, our department sequenced DNA samples from over 800 MDS bone marrow samples. During clinical follow‐up, when a patient showed potential clinical progression from MDS to sAML (secondary AML), repeated targeted sequencing was performed to explore some TRMs. Whole exome sequencing was performed when conclusive results for the last mutation events were not obtained via targeted sequencing and when adequate residual DNA extracts were available. Single‐cell RNA transcription sequencing was used to examine the underlying transformation dynamics. Diagnoses for MDS and sAML were established in strict accordance with the World Health Organization criteria, and the CMML subset were also included in this assay according to the FAB classification.^13^, ^14^ Clinical and hematologic data were recorded after informed consent was sought in accordance with the Declaration of Helsinki. This study was approved by the hospital review board of the Shanghai Jiao Tong University Affiliated Sixth People's Hospital.

### 2.2 Genomic DNA preparation, target enrichment, and sequencing

Genomic DNA (gDNA) was extracted using the DNeasy Blood and Tissue Kit (Qiagen) according to the manufacturer's protocol. Genomic DNA was sheared using the Covaris® system (Covaris), and the DNA samples were prepared using the TruSeq DNA Sample Preparation Kit (Illumina) according to the manufacturer's protocol.

Regarding the probe design, both coding and regulatory regions of target genes were included in the custom panel. The regulatory regions comprised promoter regions (defined as 2 kb upstream of the transcription start site), 5′ un‐translated region (5′‐UTR), and intron‐exon boundaries (50 bp). Custom capture oligos were designed using the SureDesign website of Agilent Technologies (Agilent). Hybridization reactions were carried out on ABI 2720 Thermal Cycler (Life Technologies) with the following hybridization conditions. The hybridization mixture was incubated for 16 or 24 h at 65°C with a heated lid at 105 °C. After the hybridization reactions, the hybridization mixture was captured and washed with magnetic beads (Invitrogen, USA) and a SureSelect target enrichment kit (Agilent, USA). The captured product was enriched with the following cycling conditions, 98 °C for 30 s, 10 cycles of 98 °C for 10 s, 60°C for 30 s, 72°C for 30 s, and 72°C for 5 min. Library quality was assessed using an Agilent 2100 Bioanalyzer (Agilent), and multiplexed sequencing was performed on HiSeq 2500 sequencers with 2 × 150 paired‐end modules (Illumina). The average sequencing depth was 800×. The following 39 sequenced target genes were included and were mainly epigenetic regulation‐related, RNA splicing‐related, and signalling, transcription, and tumour suppressor genes: AS: *ANKRD11*, *ASXL1*, *BCOR*, *CALR*, *CBL*, *CEBPA*, *DHX9*, *DNMT3A*, *ETV6*, *EZH2*, *FLT3*, *GATA2*, *IDH1*, *IDH2*, *ITIH3*, *JAK2*, *KIF20B*, *KIT*, *KRAS*, *MPL*, *NF1*, *NPM1*, *NRAS*, *PHF6*, *PTPN11*, *PTPRD*, *ROBO1*, *ROBO2*, *RUNX1*, *SETBP1*, *SF3B1*, *SRSF2*, *STAG2*, *TET2*, *TP53*, *U2AF1*, *UPF3A*, *WT1*, and *ZRSR2*.

### 2.3 Whole‐exome sequencing

The gDNA library was prepared using a TruSeq DNA Sample Preparation Kit (Illumina) in accordance with the manufacturer's protocol. In‐solution exome enrichment was performed using a TruSeq Exon Enrichment kit (Illumina) according to the manufacturer's instructions. The enriched DNA samples were sequenced via 2 × 100 paired‐end sequencing using a Hiseq2000 Sequencing System (Illumina). Illumina Sequencing Control v2.8, Illumina Off‐Line Basecaller v1.8, and Illumina Consensus Assessment of Sequence and Variation v1.8 software were used to produce 100‐base pair (bp) sequence reads.

### 2.4 Sequencing data processing, variant calling, and annotation

Before variant calling, the raw sequence reads were mapped to the reference genome (hg19). Duplicate reads were marked and removed to mitigate biases introduced by amplification, and base quality scores were recalibrated using the Genome Analysis Toolkit (GATK). The Ensembl VEP and vcf2maf tools were applied to generate a MAF format for somatic mutation annotation, and the ANNOVAR tool was used to annotate the frequency information of variations in the population database. The variants were identified as low‐frequency functional mutations if they had <0.1 frequency in the ExAC03 database, < 0.01 frequency in the 1000‐genome database, and <0.05 frequency in the GeneskyDB database. According to the results, the variant was then extracted as one of the following functional annotations: “Frame_Shift_Del/ins”, “In_Frame_ Del/Ins”, “Missense_Mutation”, “Nonsense_Mutation”, “Nonstop_Mutation”, “Splice_Site” or “Translation_Start_Site”.

### 2.5 Single‐cell RNA sequencing and bioinformatic data analysis

Bone marrow mononuclear cell concentrations were measured by haemocytometer and adjusted to 700–2000 cells/μL. Single‐cell RNA‐seq libraries were generated using 10x Genomics Single Cell 3′ reagent v2 according to the manufacturer's instructions. Twelve and 14 cycles were used for complementary DNA and index polymerase chain reaction amplifications, respectively. Amplified cDNA and library quality were assessed using Agilent 2100 Bioanalyzer (Agilent). Libraries were pooled and sequenced on the HiSeqX 10 platform (Illumina) with 2 × 150 paired‐end modules generating at least 50 K mean reads per cell. Raw sequencing data were processed using Cell Ranger version 3.1.0 (10x Genomics) with default parameters. Quality control metrics were used to remove cells with a mitochondrial gene percentage of more than 20% or cells with fewer than 200 genes detected. Variably expressed gene selection, dimensionality reduction, and clustering were performed using the Seurat package version 3.1.3. Principal component analysis was performed on significantly variable genes, and the first 40 principal components were used for UMAP dimension reduction. The cell type of each cluster was identified using singleR version 1.0.1. For paired samples, differentially expressed genes (fold change >2 and Wilcox test *p*‐value <0.05) were identified in each of the seven cell types independently, of which the mutation genes *PTPN11* and *NRAS* were used for GO and KEGG enrichment analysis.

### 2.6 Definition of the presumed transformation‐related mutation

When we analysed the paired data, if pre‐existing genes at MDS diagnosis or newly‐emerging genes at sAML transformation met the following conditions, we considered them to be sAML TRMs: 1. they must be involved in at least one of the three function pathways, namely, active signalling, myeloid transcription or tumour suppression; 2. they emerged after sAML transformation (better weight) or pre‐existed at MDS diagnosis (poorer weight). Newly emerged mutations were preferentially considered as the presumed TRMs; 3. when ≥2 suspicious mutations co‐existed to be defined as TRMs, the biologically more aggressive one (active signalling > myeloid transcription > tumour suppressor) or with lower various allele frequency (VAF) among the newly emerged mutations (meaning latest emergence) were defined as TRMs. Occasionally, more than one mutation could be presumed as TRMs.

### 2.7 Statistics analysis

Statistical analyses were conducted using SPSS software version 18.0. The Kaplan–Meier analysis was used to evaluate the time to survival and time to progression. All P‐values were based on 2‐sided tests, and *p*‐values less than 0.05 were considered statistically significant.

## 3 RESULTS

### 3.1 Targeted sequencing

Paired samples acquired from 72 patients with MDS before and after transformation to sAML were analysed using targeted sequencing. Tiers 1(hot spot mutations) and 2 (potential pathogenic but not confirmed mutation) were included in this study. The somatic mutations identified are presented in Table 1. Of course, 72 cases are much lower than the actual number of sAML‐transformed patients. In other words, because of the patients lost to follow‐up and those that rejected repeat sequencing, only some patients with sAML were repeatedly sequenced and included in our analysis.

**FIGURE 1:**
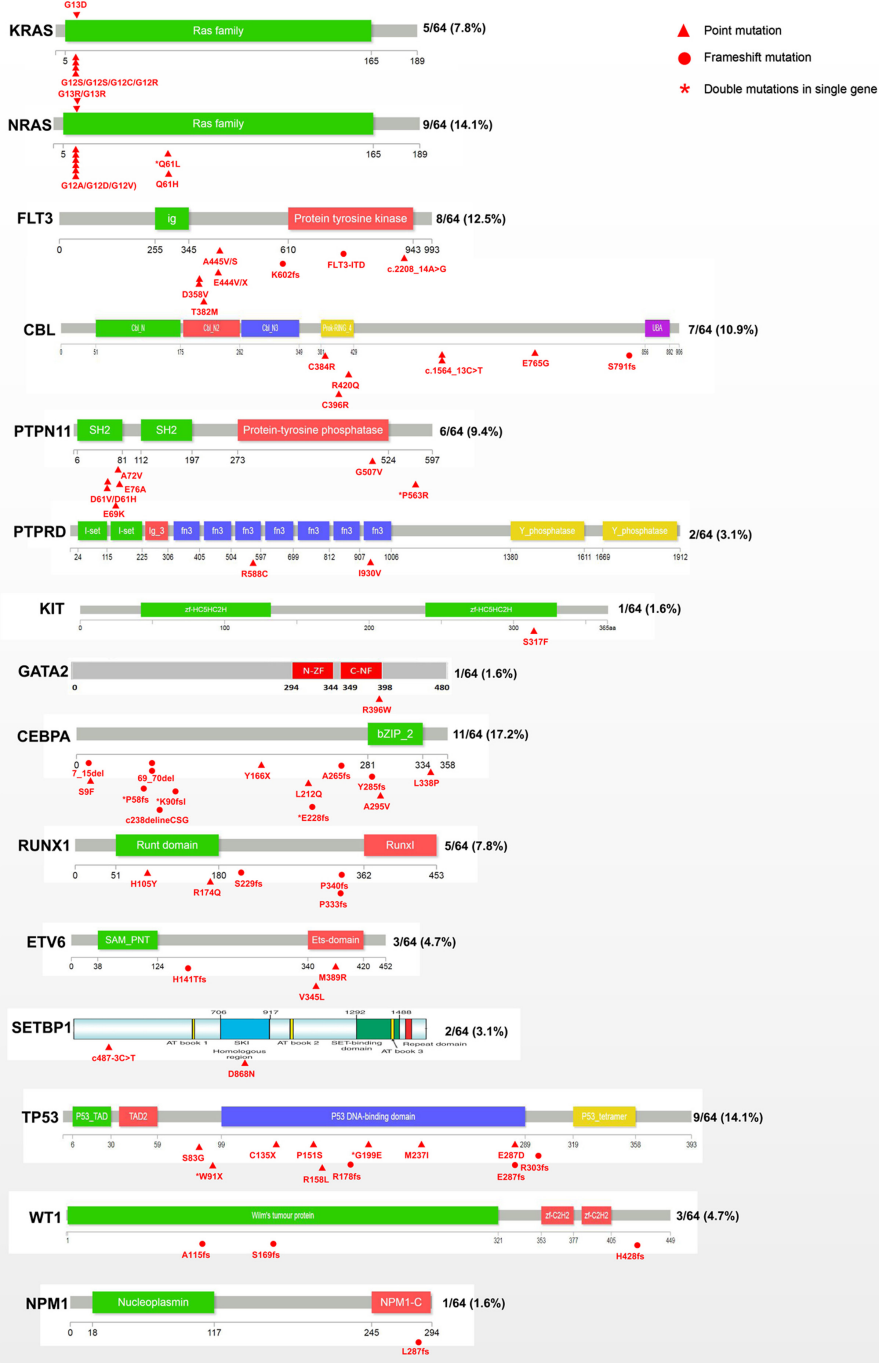
Occurrence frequency and sites of last mutation events. All mutations, including the types and sites, are shown for 13 AML TRMs. The triangles and circles indicate point and frameshift mutations, respectively. The stars indicate double mutations occurring in a single gene.

**FIGURE 2:**
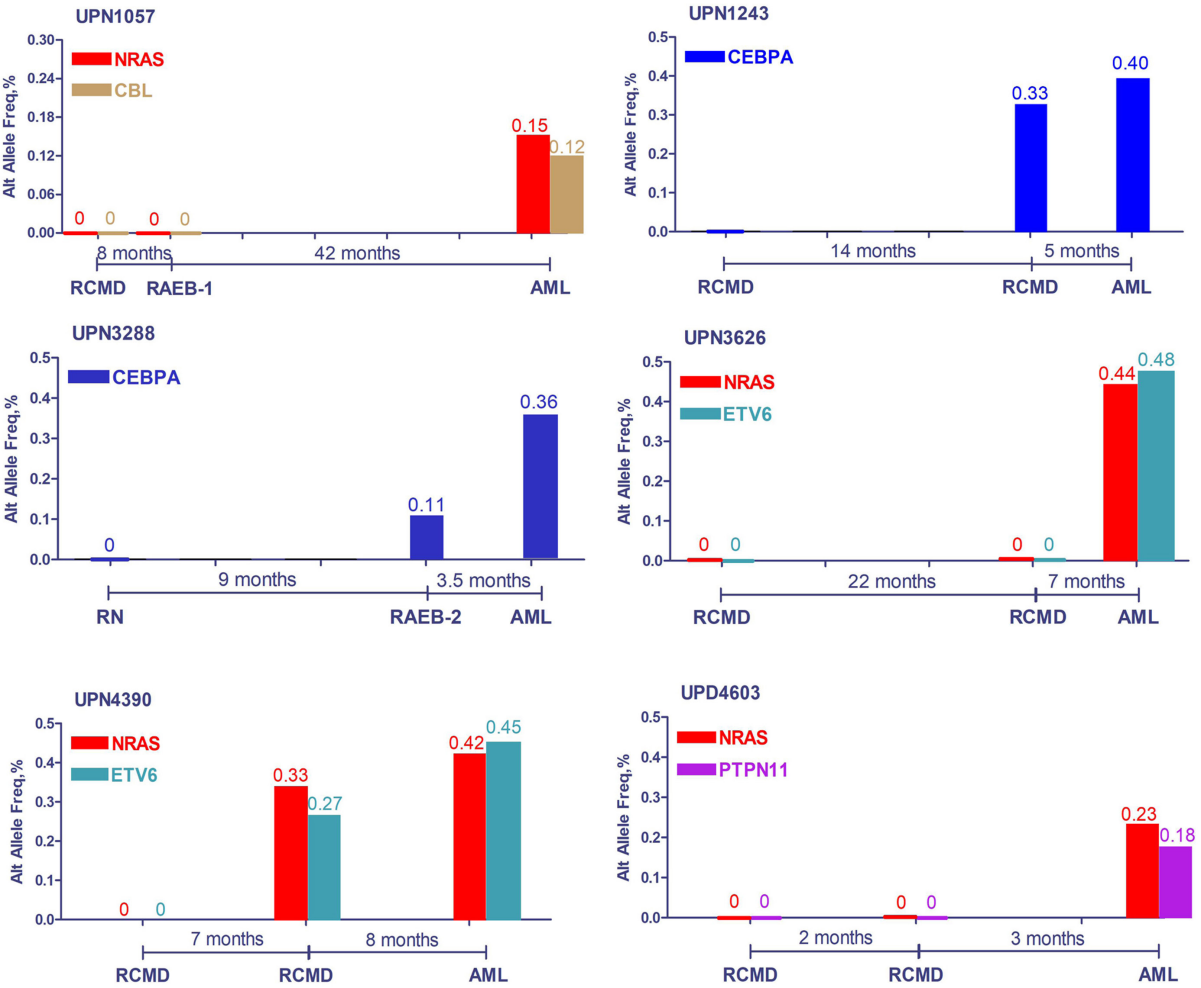
Evolution route for six cases that were analysed by an additional sequencing assay between MDS diagnosis and AML transformation.

**FIGURE 3:**
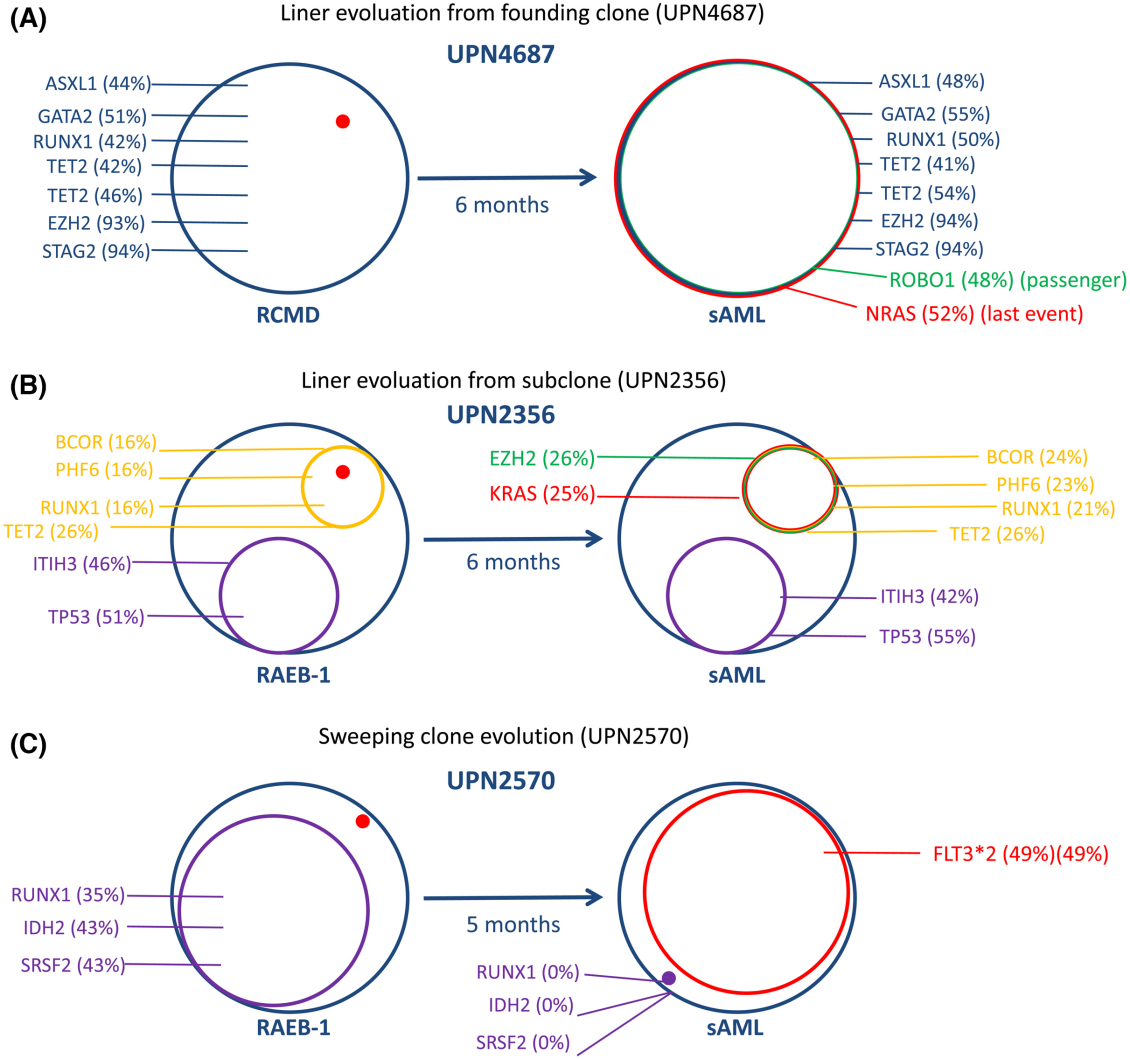
Three evolution patterns for AML transformation. (A). Linear evolution, based on the founding clone, is shown for UPN4687. Similar cases include UPN809, 1057, 3435, 3626, 1792, 3183, 1891, 1243, 3288, 1090, 1018, 3552, 3826, 4196, 4634, 1582, 1831, 2296, 843, 702, 3430, 3541, 2638, 4133, 4820, 4877, 5135 and 5305. (B). Linear evolution from a subclone is shown for UPN2356. Similar cases include UPN4603, 2577, 692, and 2666. Linear evolution includes two models (from founding clone and subclone). Red dots mean the origin of the red cycle (right graph). (C). Sweeping clone evolution is shown for UPN2570. Similar cases include UPN811, 1354, 3053, 3567 and 4383. [Number] indicates VAF; [H] indicates homozygous mutations; red indicates presumed the last mutation; green indicates newly emerged partner mutations, which could not be presumed to be AML TRMs.

1. TRMs were identified in 64 of the 72 patients (88.9%), according to the steps described in the Methods section.
2. Among these 64 patients, TRMs were detected only at AML transformation in 40 patients (representing 62.5% of these cases). In the 24 remaining cases, these mutations were detected at the point of MDS diagnosis (Table 1). Patients with TRMs in only their sAML sample trended toward a longer time duration to undergo AML transformation (median 11.5 months vs. 8 months, *p* = 0.045), but without a significant difference in the frequency of lower risk (<RAEB2/CMML2) cases (77.5% vs. 75.0%, *p* = 0.819).
3. The total defined TRM number was more than 64 because sometimes, more than one mutation could be considered a TRM for a patient, and occasionally the TRMs for one case could be related to both signalling and transcription. As seen in Tables 1 and 2 and Figure 1, the following defined TRMs from the 64 patients were present in descending order of frequency. The first were active signalling mutations: *KRAS*/*NRAS* in 14 patients; *FLT3* in eight patients; *CBL* in seven patients; *PTPN11* in six patients; *PTPRD* in two patients; *KIT* and *GATA2* in one patient, respectively. The next set of mutations comprised myeloid transcription factors: *CEBPA* in 11 patients; *RUNX1* in five patients; *ETV6* in three patients; *SETBP1* in two patients, respectively. The next set of mutations comprised tumour suppressor genes: *TP53* mutations in nine patients; *WT1* mutations in three patients; *NPM1* in one patient. The TRM events seemed to be highly enriched in seven genes. Figure 1 demonstrated that the involvement style of *FLT3* (seven of the eight cases appeared to be point mutations) was not the same as that of *FLT3‐ITD*, which is more often observed in de novo AML.
4. In six of the 64 paired samples with candidate transformation events, we sequenced an additional sample between MDS diagnosis and AML transformation (Figure 2). Of these, three paired samples (UPN1243, 3288, and 4390) showed evidence of the TRMs emerging before phenotype change, that is, the TRMs event emerged while the disease was still in the MDS stage, with sAML transformation occurring soon after.
5. An analysis of the changing mutation profiles and VAF, as well as the logical relations between them, revealed differing patterns of evolution from MDS to sAML. Of the 64 patients with candidate mutation events, most (29 cases) demonstrated AML‐transformation‐related progression by linear evolution from the founding clone (Figure 3A). Five cases showed linear evolution from subclones (Figure 3B). Six cases demonstrated sweeping clonal evolution (Figure 3c). The evolution pattern of the remaining 24 patients either could not be defined by changes in mutations or VAF (four cases) or harboured no candidate alterations before or after progression to AML.
6. Rivalry clonal evolution patterns were observed. Some original TRMs were replaced or outgrown by other mutations at sAML transformation, such as in patient 1 (UPN809); 6 (UPN3430), 10 (UPN4603), 18 (UPN2570), and 63 (UPN3567) (Table 1).
7. Although differences existed among TRMs, *TET2/RUNX1/ASXL1/ DNMT3A/U2AF1/STAG2/ROBO1/SF3B1* mutations were the most common initial/driver mutations in the AML‐transformed patients included (Table 2). However, A*SXL1/BCOR/TET2* mutations were most commonly accompanied by transformation‐related events when the transformation occurred (Table 2).

**TABLE 1:** Results of the targeted sequencing from the paired samples of 72 patients

No(UPN)	Diagnosis	Kary otype	leukaemia‐free survival (m)	Presumed last event	All gene mutations
*The patients in whom AML transformation‐related mutations could be presumed by paired sequencing analysis* (*listed as signalling pathway activation*, *transcription factors and tumour suppressors* signalling pathway activation					
1.(809)	RCMD	normal		*CEBPA/1/K352fs*(*15*) *FLT3/9/D358V*(*48*) *RUNX1/6/508 + 1G > C*(*24*)*/5/G170R*(*23*)	*ASXL1*; *CEBPA*; *EZH2*; *FLT3*; RUNX1*2; *STAG2*; *TET2*
	AML	normal	17	*FLT3/9/D358V*(*48*) *RUNX1/6/508 + 1G > C*(*45*)*/5/G170R*(*42*) ***NRAS/2/G13R*(*48*)**	*ASXL1*; *EZH2*; *FLT3*; *RUNX1 × 2*; *STAG2*; *TET2*; *BCOR*; *NRAS*
2.(1057)	RAEB1	normal		*PTPRD/10/A539V*(*51*)	*DNMT3A*; *PTPRD*; *STAG2*; *U2AF1*
	AML	normal	48	*PTPRD/10/A539V*(*46*) *RUNX1/4/L98fs*(*28*) ***NRAS/2/3/G12V/Q61L*(*15*) (*15*)** ***CBL/8/C384R*(*12*)**	*DNMT3A*; *PTPRD*; *STAG2*; *U2AF1*; *CBL*; *NRAS×2*; *RUNX1*
3.(2356)	RAEB1	normal		*PHF6/4/C107fs*(*16*) *RUNX1/5/A149_A150delinsGX*(*16*) *TP53/3/c.74 + 14 T > C*(*51*)	*BCOR*; *ITIH3*; *PHF6*; *RUNX1*; *TET2*; *TP53*
	AML	normal	6	*PHF6/4/C107fs*(*23*) *RUNX1/5/A149_A150delinsGX*(*21*) *TP53/3/c.74 + 14 T > C*(*55*) ***KRAS/2/G13D*(*25*)**	*BCOR*; *ITIH3*; *PHF6*; *RUNX1*; *TET2*; *TP53*; *EZH2*; *KRAS*
4.(2998)	RAEB2	normal		*RUNX1/9/967 + 1 > A6/886 + 1 > A*(*31*) *KRAS/2/G12S*(*30*)	*ASXL1*; *KRAS*; *RUNX1*; *U2AF1*
	AML	normal	2	*RUNX1/9/967 + 1 > A6/886 + 1 > A*(*28*) ***KRAS/2/G12S*(*27*)**	*ASXL1*; *KRAS*; *RUNX1*; *U2AF1*
5.(2747)	RAEB2	normal			*BCOR*; *ITIH3*
	AML	normal	10	***NRAS/2/G12D*(*57*)**	*BCOR*; *ITIH3*; *KIF20B*; *NRAS*; *STAG2*;
6.(3430)	CMML1	normal		*GATA2/4/G327E*(*29*)*/4/323_323del*(*13*) *RUNX1/6/D198Y*(*41*) *KRAS/2/G12C*(*11*) *NRAS/2/G12D*(*29*)	GATA2*2; *KRAS*; *NRAS*; *RUNX1*; *SF3B1*; *UPF3A*
	AML	normal	7	*GATA2/4/G327E*(*29*)*/4/323_323del*(*38*) *RUNX1/6/D198Y*(*43*) ***KRAS/2/G12C*(*45*)**	GATA2*2; *KRAS*; *RUNX1*; *SF3B1*; *UPF3A*; *EZH2*
7.(3435)	RCMD	normal		*RUNX1/1/P86S*(*46*)	*ASXL1*; *EZH2*; *RUNX1*; *STAG2*; *UPF3A*
	AML	normal	33	*RUNX1/1/P86S*(*43*) *PTPN11/3/F71L*(*34*) ***NRAS/2/G12D*(*9.7*)**	*ASXL1*; *EZH2*; *RUNX1*; *STAG2*; *UPF3A*; *BCOR*; *NRAS*; *PTPN11*
8.(3541)	RAEB2	del20q‐		*GATA2/5/L345W*(*43*)	*DNMT3A*; *GATA2*; *TET2*
	AML	del20q‐	14	*GATA2/5/L345W*(*30*) ***KRAS/2/G12R*(*30*)**	*DNMT3A*; *GATA2*; *TET2*; *BCOR*; *KRAS*
9.(3626)	RCMD	normal			*ASXL1*; *DHX9*; *IDH1*; *RUNX1*; *TET2*; *U2AF1*
	AML	NA	29	*ETV6/6/G381fs*(*48*) ***NRAS/2/G12A*(*44*)**	*ASXL1*; *DHX9*; *IDH1*; *RUNX1*; *TET2*; *U2AF1*; *ETV6*; *NRAS*
10.(4674)	RCMD	Complex		*RUNX1/1/H78Y*(*53*) *ETV6/6/R339I*(*15*)	*DNMT3A*; *ETV6*; *EZH2*; *KIF20B*; *RUNX1*; *SETBP1*
	AML	Complex	5	*RUNX1/1/H78Y*(*43*) ***NRAS/2/G12A*(*23*)** ***PTPN11/3/A72V*(*18*)**	*DNMT3A*; *KIF20B*; *RUNX1*; *SETBP1*; *NRAS*; *PTPN11*;
11.(4687)	RCMD	normal		*GATA2/3/M223I*(*51*) *RUNX1/2/G138fs*(*42*)	*ASXL1*; *EZH2*; *GATA2*; *RUNX1*; *STAG2*; TET2*2
	AML	NA	6	*GATA2/3/M223I*(*55*) *RUNX1/2/G138fs*(*50*) ***NRAS/2/G12D*(*52*)**	*ASXL1*; *EZH2*; *GATA2*; *RUNX1*; *STAG2*; TET2*2; *ROBO1*; *NRAS*
12.(4877)	RCMD	normal		*MPL/2/A58V*(*47*) *KRAS/2/G12S*(*41*)	*ASXL1*; *KRAS*; *MPL*; *STAG2*; *TET2*;
	AML	normal	17	*MPL/2/A58V*(*46*) ***KRAS/2/G12S*(*55*)** ***PTPRD/23/R588C*(*52*)**	*ASXL1*; *KRAS*; *MPL*; *STAG2*; *TET2*; *PTPRD*
13.(5305)	RCMD	normal		*NRAS/2/G13D*(*4.1*)*/2/G12D*(*3.4*)	*IDH1*; *KMT2D*; *NRAS*;
	AML	normal	9	***NRAS/2/G13D*(*38*)** ***ETV6/4/H141Tfs313*(*16*)**	*IDH2*; *KMT2D*; *NRAS*; *ETV6*
14.(5253)	RCMD	normal		*None*	*None*
	AML	t(3; 6), −7	40	*RUNX1/6/R201Q*(*48*) ***NRAS/3/Q61H*(*25*)** ***PTPN11/3/E69K*(*23.4*)**	*ANKRD11*; *NRAS*; *PTPN11/RUNX1*
15.(1587)	RAEB1	der2		*JAK2/14/V617F*(*46*) *FLT3/19/c.2208‐14A.G*(*52*)	*IDH2*; *FLT3*; *JAK2*; *U2AF1*; *ZRSR2*
	AML	der2	25	*JAK2/14/V617F*(*43*) ***FLT3/19/c.2208‐14A.G*(*49*)**	*IDH2*; *FLT3*; *JAK2*; *U2AF1*; *ZRSR2*
16.(1792)	RAEB1	der4		*RUNX1/9/Q397R*(*47*)	*RUNX1*; *U2AF1*
	AML	der4	8	*RUNX1/9/Q397R*(*30*) ***FLT3/11/E444V*(*37*)*11/E444X*(*37*)**	*RUNX1*; *U2AF1*; FLT3*2; *ROBO1*;
17.(2117)	RCMD	complex		*TP53/5/8/Y126C*(*16*)*/R273S*(*15*)	*SRSF2*; TP53*2
	AML	complex	8	*TP53/5/8/Y126C*(*14*)*/R273S*(*14*) ***FLT3/9/D358V*(*55*)**	TP53*2; *FLT3*
18.(2570)	RAEB1	normal		*RUNX2/6/D198N*(*35*)	*IDH2*; *RUNX1*; *SRSF2*
	AML	+8	5	***FLT3/11/A445V*(*49*)*/11/A445S*(*49*)**	FLT3*2;
19.(2577)	CMML1	normal		*CEBPA/1/p69‐70del*(*17*) *NPM1/10/L258fs*(*33*)	*CEBPA*; *DNMT3A*; *NPM1*; *ROBO1*; *TET2*
	AML	normal	11	*CEBPA/1/p69‐70del*(*21*) *NPM1/10/L258fs*(*25*) ***FLT3/14/K602delinsREYEYDLK* (*14*)**	*CEBPA*; *DNMT3A*; *NPM1*; *ROBO1*; *TET2*; *FLT3*
20.(3183)	RCMD	add (3) + 8			*BCOR*; *DHX9*; EZH2*2; *IDH1*; *ROBO1*; *STAG2*; *U2AF1*
	AML	add (3) + 8	6	***FLT3/9/D358V*(*52*)** ***CBL/11/C1564‐13C > T*(*40*)**	*BCOR*; *DHX9*; EZH2*2; *IDH1*; *ROBO1*; *STAG2*; *U2AF1*; *CBL*; *FLT3*; *TET2*
21.(4383)	RCMD	normal			*DNMT3A*; *SF3B1*
	AML	+1, der(1; 16)	18	*PHF6/2/S22★*(*5.6*) ***FLT3/9/T382M*(*45*)** ***CBL/15/E765G*(*49*)** ***KIT/14/S713F*(*49*)**	*DNMT3A*; *SF3B1*; *CBL*; *FLT3*; *KIT*; *PHF6*
22.(5135)	RAEB2	Add7, del20q			*ASXL1*; *ITIH3*; *TET2*; *U2AF1*
	AML	Add7, del20q	5	***FLT3‐ITD*(*high*)*1.597***	*ASXL1*; *ITIH3*; *TET2*; *U2AF1*; *FLT3‐ITD*
23.(692)	CMML2	normal		*GATA2/3/S186T*(*47*) *PHF6/9/:c.968 + 1G > A*(*25*) *RUNX1/9/F416fs*(*27*)	*GATA2*; *ITIH3*; *PHF6*; *RUNX1*; *SF3B1*;
	AML	normal	10	*GATA2/3/S186T*(*50*) *PHF6/9/:c.968 + 1G > A*(*26*) *RUNX1/9/F416fs*(*24*) ***CBL/9/R420Q*(*34*)**	*GATA2*; *ITIH3*; *PHF6*; *RUNX1*; *SF3B1*; *CBL*
24.(1891)	CMML1	der22			*DNMT3A*; *ROBO2*; *TET2*
	AML	der22	14	***CBL/11/c.1564‐13C > T*(*56*)** ***CEBPA/1/A265fs*(*37*)*K90fs*(*52*)**	*DNMT3A*; *ROBO2*; TET2*2; *CBL*; CEBPA*2; *EZH2*
25. (2206)	RCMD	+8		*NF1/17/M645V*(*49*) *PTPRD/29A1047S*(*44*) *CBL/8/C396R*(*27*)	*ANKRD11*; *ASXL1*; *CBL*; *NF1*; *PTPRD*; *U2AF1*
	AML	+8	3	*NF1/17/M645V*(*51*) *PTPRD/29A1047S*(*40*) ***CBL/8/C396R*(*21*)**	*ANKRD11*; *ASXL1*; *CBL*; *NF1*; *PTPRD*; *U2AF1*
26.(4559)	RAEB2	complex		*CBL/15/S791fs*(*9.6*)*/15/L790fs*(*9.7*)	*ANKRD11*; *ASXL1*; CBL*2; *ROBO1*
	AML	complex	11	***CBL/15/S791fs*(*8.7*)*/15/L790fs*(*8.8*)**	*ANKRD11*; *ASXL1*; CBL*2; *ROBO1*
27.(2638)	CMML1	normal		*PTPN11/14/P563R*(*43*)	*PTPN11*; *ROBO1*;
	AML	normal	16	***PTPN11/14//P563R*(*45*) */3/D61V*(*37*)**	*PTPN11 × 2*; *ROBO1*; *SF3B1*
28.(4133)	RAEB1	complex		*CBL/9/R420Q*(*71*) *TP53/4/c.306‐2A > G*(*96*)	*ASXL1*; *CBL*; *ROBO2*; *TP53*
	AML	complex	3	*CBL/9/R420Q*(*39*) *TP53/4/c.306‐2A > G*(*97*) ***PTPN11/13/G507V*(*28*)**	*ASXL1*; *CBL*; *ROBO2*; *TP53*; *PTPN11*
29.(4410)	RAEB1	del20q		*PTPN11/3/D61H*(*33*)	*EZH2*; *PTPN11*; *SETBP1*; *TET2*
	AML	del20q	12	***PTPN11/3/D61H*(*48*)**	*EZH2*; *PTPN11*; *SETBP1*; *TET2*
30.(4820)	RCMD	Add3, add18			*EZH2*; *KMT2D*; *U2AF1*
	AML	Add3	15	***PTPN11/3/E76A*(*8.0*)**	*EZH2*; *KMT2D*; *U2AF1*; *PTPN11*
31.(3891)	RCMD	normal		*PTPRD/18/I930V*(*51*)	*ASXL1*; *PTPRD*
	AML	normal	8	***PTPRD/18/I930V*(*46*)**	*ASXL1*; *PTPRD*
32.(702)	RCMD	der16, (1; 16) del20q			*ANKRD11*; *SRSF2*
	AML	der16t (1; 16) del20q	28	*MPL/10/W515L*(*17*) ***GATA2/6/R396W*(*15*)**	*ANKRD11*; *SRSF2*; *GATA2*; *IDH1*; *MPL*
*Myeloid transcriptors*					
33.(1018)	RAEB2	derX, der14			*DNMT3A*; *EZH2*; *TET2*
	AML	derX, der14	4	*FLT3:/9/D358V*(*53*) ***CEBPA/1/A295V*(*39*)**	*DNMT3A*; *EZH2*; *TET2*; *BCOR*; *CEBPA*; *FLT3*
34.(1090)	RARS	del20q		*RUNX1/9/D344G*(*45*)	*DNMT3A*; *DHX9*; *RUNX1*; *SRSF2*;
	AML	del20q	11	*RUNX1/9/D344G*(*48*)*/7/R232W*(*20*) ***CEBPA/1/Y285fs*(*32*)**	*DNMT3A*; *DHX9*; RUNX1*2; *SRSF2*; *ASXL1*; *CEBPA*; *SETBP1*
35.(1243)	RCMD	normal			*ASXL1*; *U2AF1*
	AML	normal	18	***CEBPA/1/7_15del*(*40*)**	*ASXL1*; *U2AF1*; *CEBPA*; *IDH1*
36 (1931)	RAEB1	complex		***CEBPA/1/S9F*(*67*)**	*CEBPA*; *DNMT3A*; *TET2*; *U2AF1*
	AML	complex	9	***CEBPA/1/S9F*(*46*)**	*CEBPA*; *DNMT3A*; *TET2*; *U2AF1*
37.(3288)	RN	normal			*KIF20B*; *STAG2*; TET2*2
	AML	normal	12	***CEBPA/1/L338P*(*36*)**	*KIF20B*; *STAG2*; TET2*2; *ASXL1*; *CEBPA*; *EZH2*
38.(3552)	RARS	‐Y			*SF3B1*; *TET2*
	AML	NA	28	***CEBPA/1/c238delineCSG*(*18*)**	*SF3B1*; *TET2*; *CEBPA*; *IDH2*; *SRSF2*; *STAG2*
39.(3826)	RCMD	del7			*ASXL1*; *EZH2*
	AML	normal	22	***CEBPA/1/Y166X*(*48*)*P58fs*(*47*)**	*ASXL1*; *EZH2*; CEBPA*2
40.(4196)	RCMD	del20q, +21		*PHF6/4/G93S*(*27*) *RUNX1/1/R80C*(*51*)	*PHF6*; *RUNX1*; *SRSF2*;
	AML	del20q, +21	8	*PHF6/4/G93S*(*45*) *RUNX1/1/R80C*(*78*) *NRAS/2/G12D*(*34*) ***CEBPA/1/69_70del*(*11*)**	*PHF6*; *RUNX1*; *SRSF2*; *CEBPA*; *NRAS*
41.(4634)	RAEB2	normal		*NPM1/7/L160fs*(*24*) *NRAS/2/G13D*(*16*)	*NPM1; NRAS; SF3B1*; *TET2*
	AML	normal	2	*NPM1/7/L160fs*(*35*) *NRAS/2/G13D*(*37*) ***CEBPA/1/69_70del*(*9.8*)**	*NPM1; NRAS; SF3B1*; *TET; CEBPA*
42.(4806)	CMML1	normal			*ANKRD11*; *DHX9*; *TET2*;
	AML	+8	11	***CEBPA/1/1/E228fs*(*4.8*)*/L212Q*(*28*)**	*ANKRD11*; *DHX9*; *TET2*; *CEBPAX2*
43.(1350)	RAEB1	complex		*PHF6/9/Y303X*(*56*) *RUNX1/9/P333fs*(*29*)	*EZH2*; *PHF6*; *RUNX1*; *TET2*
	AML	complex	2	*PHF6/9/Y303X*(*46*) ***RUNX1/9/P333fs*(*17*)**	*EZH2*; *PHF6*; *RUNX1*; *TET2*
44.(1582)	CMML1	normal		*PTPRD/22/c.962‐9C > G*(*47*)	*KIF20B*; *PTPRD*; *SF3BA*; *TET2*
	AML	normal	13	*PTPRD/22/c.962‐9C > G*(*43*) ***RUNX1/9/P340fs*(*40*)**	*KIF20B*; *PTPRD*; *SF3BA*; *TET2*; *RUNX1*
45.(3479)	RCMD	normal		*RUNX1/3/R174Q*(*28*)	*ASXL1*; *RUNX1*; *TET2*; *ZRSR2*
	AML	normal	15	***RUNX1/3/R174Q*(*42*)**	*ASXL1*; *RUNX1*; TET2*2; *ZRSR2*
46.(3536)	RCMD‐RS	normal		*RUNX1/4/H105Y*(*40*)	*ASXL1*; *ROBO1*; *RUNX1*; *SF3B1*; *UPF3A*
	AML	del5q	3	***RUNX1/4/H105Y*(*34*)**	*ASXL1*; *ROBO1*; *RUNX1*; *SF3B1*; *UPF3A*
47.(4143)	RAEB1	normal		*RUNX1/4/S229fs*(*34*)	*BCOR*; *DNMT3A*; *IDH2*; *ROBO1*; *RUNX1*
	AML	normal	3	***RUNX1/4/S229fs*(*39*)**	*BCOR*; *DNMT3A*; *IDH2*; *ROBO1*; *RUNX1*
48.(811)	RCMD	der6, t (1;6)			*None*
	AML	der6, t (1;6)	26	***ETV6/6/V345L*(*52*)** ***MPL/10/W515R*(*50*)**	*ETV6*; *MPL*
49.(4390)	RCMD	normal		*ETV6/7/M389R*(*33*) *PHF6/10/R342X*(*27*)	*ASXL1*; *BCOR*; *ETV6*; *PHF6*
	AML	inv3add3	15	*PHF6/10/R342X*(*45*) ***ETV6/7/M389R*(*42*)**	*BCOR*; *ETV6*; *PHF6*
50.(1601)	RAEB2	normal		*SETBP1/3/c487‐3C > T*(*44*)	*SETBP1*
	AML	normal	8	***SETBP1/3/c487‐3C > T*(*43*)**	*SETBP1*
51.(3053)	RAEB2	normal			*STAG2*; *UPF3A*
	AML	normal	40	***SETBP1/4/D868N*(*18*)**	*SETBP1*; *SRSF2*
*Tumour suppressors*					
52.(843)	RCMD	der1del1			*SRSF2*; *TET2*
	AML	der1	3	***TP53/5/C135X*(*18*)**	*SRSF2*; *TET2*; *TP53*
53.(1354)	RAEB1	normal			*None*
	AML	complex	7	***TP53/8/E287fs*(*40*)*/6/G199E*(*28*)**	*ASXL1*; *TP53 × 2*
54.(1445)	RAEB2	normal		*TP53/8/E287D*(*56*)	*ROBO1*; *TP53*; *UPF3A*
	AML	del11q	27	***TP53/8/E287D*(*48*)**	*ROBO1*; *TP53*; *UPF3A*
55.(1694)	RAEB2	complex		*TP53/7/M237I*(*74*)	*DNMT3A*; *TP53*
	AML	complex	3	***TP53/7/M237I*(*80*)**	*DNMT3A*; *TP53*
56.(1831)	RAEB1	complex		*TP53/4/W91X*(*30*)	*ANKRD11*; *DNMT3A*; *TP53*
	AML	complex	13	***TP53/4/W91X*(*30*)*/5/R158L*(*31*)**	*ANKRD11*; *DNMT3A*; TP53*2; *KIF20B*; *STAG2*
57.(2296)	RAEB2	normal			*DHX9*; *ROBO1*
	AML	normal	4.5	***TP53/9/R303fs*(*85*)**	*DHX9*; *ROBO1*; *TP53*
58.(2666)	RAEB1	normal		*PTPRD/27/G809V*(*56*)	*DNMT3A*; *IDH2*; *PTPRD*
	AML	normal	18	*PTPRD/27/G809V*(*51*) ***TP53/5/P151S*(*25*)**	*DNMT3A*; *IDH2*; *PTPRD*; *TP53*
59.(3678)	RAEB1	complex		*TP53/5/530‐535del* (*34*)	*TP53*
	AML	complex	15	***TP53/5/530‐535del*(*53*)**	*TP53*
60.(3380)	CMML1	complex		*TP53/2/S83G*(*84*)	*TP53*
	AML	complex	13	***TP53/2/S83G*(*60*)**	*TP53*
61.(1670)	RCMD	del18q		*GATA2/5/M388fs*(*41*) *TP53/2/E11Q*(*51*) *ETV6/5/V285M*(*49*) *WT1/1/A115fs*(*12*)	*BCOR*; *ETV6*; *GATA2*; *TET2*; *TP53*; *U2AF1*; *WT1*
	AML	del18q	2	*GATA2/5/M388fs*(*40*) *TP53/2/E11Q*(*50*) *ETV6/5/V285M*(*53*) ***WT1/1/A115fs*(*25*)**	*BCOR*; *ETV6*; *GATA2*; *TET2*; *TP53*; *U2AF1*; *WT1*
62.(1849)	RCMD	normal		*WT1/7/H428fs*(*31*)	*DNMT3A*; *IDH1*; *TET2*; *WT1*
	AML	normal	8	***WT1/7/H428fs*(*40*)**	*DNMT3A*; *IDH1*; TET2*2; *WT1*
63.(3567)	RAEB2	normal		*FLT3/12/c.1419–3‐ > T*(*15*) *NPM1/10/R262fs*(*43*)*/10W259delinsWQ*(*44*)*/10/R262S*(*44*)*/7/W163L*(*42*)	*DHX9*; *DNMT3A*; *ANKRD11*; *FLT3*; NPM*4
	AML	normal	4	*NPM1/10/R262fs*(*12*)*/10W259delinsWQ*(*25*)*/10/R262S*(*19*)*/7/W163L*(*20*) ***WT1/7/S169fs*(*32*)**	*DHX9*; *DNMT3A*; *ANKRD11*; *NPM×4*; *ROBO1*; *WT1*
64.(1892)	RAEB2	normal		*NPM1/11/L287f*(*35*)	*DNMT3A*; *IDH1*; *NPM1*
	AML	normal	9	***NPM1/11/L287f*(*31*)**	*DNMT3A*; *IDH1*; *NPM1*
*The patients in whom last gene events could not be presumed by paired targeted sequencing analysis*					
65.(1075)	RA	normal			*DHX9*; *IDH2*; *ROBO2*; *STAG2*; *UPF3A*
	AML	normal	20		*DHX9*; *IDH2*; *ROBO2*; *STAG2*; *UPF3A*; *TET2*
66.(1619)	RAEB2	complex			*DHX9*; *DNMT3A*; ITIH3*2
	AML	complex	25		*DHX9*; *DNMT3A*; ITIH3*2
67.(1702)★	RAEB2	normal			*SRSF2*
	AML	der12	19		*SRSF2*
68.(3831) ★	RAEB2	normal			*ANKRD11*; *IDH2*
	AML	normal	4		*ANKRD11*; *IDH2*
69.(3155) ★	RCMD	+14, del20q			*ROBO1*
	AML	normal	7.5		*ROBO1*
70.(3437)	RA	Inv9			*DNMT3A*; *ROBO1*
	AML	Inv9	3		*DNMT3A*; *ROBO1*
71.(4145)	RCMD	+1, der(1:7)			*EZH2;IDH1*
	AML	NA	14		*ASXL1*; TET2*2; *ZRSR2*
72.(4404)	RAEB1	complex			*SRSF2*; *TET2*
	AML	complex	18		*TET2*

*Note*: 1. The fifth column indicates the possible last event mutations. The mutations listed in *italic* and bold font are considered the final presumed TRMs according to the definition in the Method section. 2. The paired targeted sequencing analysis from the last eight patients could not define the TRMs, so three (with star signals) of them agreed to WES to look for possible TRMs. For the explanation of mutation type, splicing site mutation was shown in nucleotide variation. 3. In column 5, NRAS/2/G13R(48) means exon2, VAF was 48%.

Abbreviations: AML, acute myeloid leukaemia; CMML1/2, chronic myelomonocytic leukaemia 1/2; NA means no available data; RA, refractory anaemia; RAEB‐1/2, refractory anaemia with excess blasts‐1/2; RARS, refractory anaemia with ring sideroblasts; RCMD, refractory cytopenias with multilineage dysplasia.

**TABLE 2:** Summary of the characteristics of the top seven TRMs by targeted sequencing

TRMs	cases	Emerged at sAML (*n*)	Primary diagnosis≥ RAEB2/CMML2 (*n*)	Most common earlier mutations at MDS diagnosis (top 2–3) (top5 in Total) (*n*)	Most common partner mutations at transformation (top 1) (top2 in Total) (*n*)
NRAS/KRAS	14(5)	10/14	3/14	*RUNX1*(*8*)*/ASXL1*(*6*)*TET2*(*6*)*STAG2*(*5*)	*BCOR*(*3*)
CEBPA	11(1)	10/11	2/11	*TET2*(*7*)*/DNMT3A*(*4*)*/ASXL1*(*2*)*EZH2*(*2*)*SF3B1*(*2*)*RUNX1*(*2*)*SRSF2*(*2*)*U2AF1*(*2*)	*ASXL1*(*2*)*EZH2*(*2*)
TP53	9	5/9	3/9	*DNMT3A*(*3*)*/ROBO1*(*2*)	*ASXL1*(*1*)*KIF20B*(*1*)*STAG2*(*1*)
FLT3	8(2)	7/8	1/8	*U2AF1*(*4*)*/IDH2*(*2*)*RUNX1*(*2*)*SRSF2*(*2*)*DNMT3A*(*2*)*ROBO1*(*2*)*TET2*(*2*)	*ROBO1*(*1*)*TET2*(*1*)
CBL	7(3)	5/7	2/7	*DNMT3A*(*3*)*/U2AF1*(*3*)*/ANKRD11*(*2*)*ASXL 1*(*2*)*STAG2*(*2*)*/PTPRD*(*2*)*/ROBO1*(*2*)*/SF3B1*(*2*)	*EZH2*(*1*)*/RUNX1*(*1*)*/PHF6*(*1*)*/TET2*(*1*)
PTPN11	6(2)	4/6	0/6	*EZH2*(*3*)*SETBP1*(*2*)	*SF3B1*(*1*)
RUNX1	5	1/5	0/5	*TET2*(*3*)*/ASXL1*(*2*)*SF3B1*(*2*)	*TET2*(*1*)*/MPL*(*1*)
Total	60(13)	42/60	11/60	*TET2*(*18*)*/RUNX1*(*12*)*/ASXL1*(*12*)*DNMT3A*(*12*)*/U2AF1*(*9*)*/STAG2*(*7*)*ROBO1*(*6*)*SF3B1*(*6*)	*ASXL1*(*3*)*BCOR*(*3*)*TET2*(*3*)

*Note*: 1. The other TRMs (≤ 3 cases in total) are not included in this table. 2. The numbers in the brackets in the second column represent the cases that had other presumed TRMs besides the mainly presumed TRMs. 3. The mutations listed in the last column refer to the mutations which emerged together with the presumed TRMs and may play a role during the MDS‐sAML transformation.

### 3.2 Whole‐exome sequencing

**FIGURE 4:**
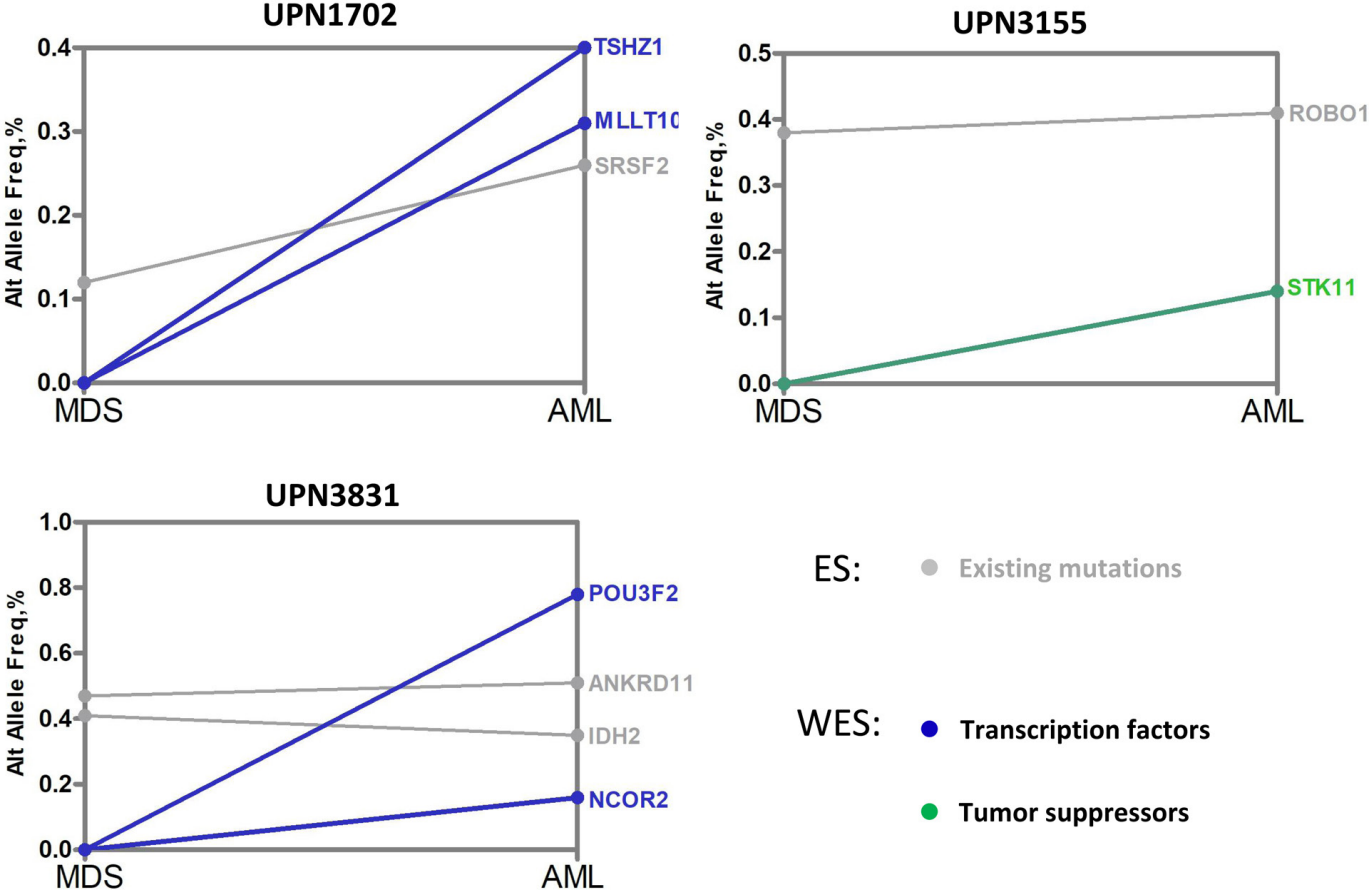
Whole‐exome sequencing revealed other atypical last mutation events. Three patients showed negative results for typical AML TRMs. However, whole‐exome sequencing revealed some other atypical AML TRMs. Grey indicates existing mutations; blue indicates transcription factors; green indicates tumour suppressors.

Samples from three of the eight patients whose targeted sequencing showed no presumed TRMs but for whom sufficient DNA extract was still available were further subjected to whole exome sequencing (WES) (patients starred [*] in Table 1). Figure 4 presents the results of WES from these cases. In addition to MDS‐related gene mutations, each gained at least one TRM (involving transcription factor or tumour suppressor genes) at the AML transformation point. Specifically, UPN1702 and UPN3831 acquired *MLLT10* and *NCOR2* mutations, respectively (both involved in transcription regulation), at the AML stage. *MLLT10* is a histone lysine methyltransferase that participates in AML pathogenesis via the formation of the fusion gene *MLLT10‐MLL*.^15^ *NCOR2* regulates gene transcription as a part of the histone deacetylases complex, and its dysfunction leads to functional abnormality in haematopoietic stem cells.^16^ UPN3155 acquired an *STK11* mutation (a tumour suppressor) after disease progression. It has been reported that *STK11* regulates cell polarity and functions as a tumour suppressor.^17^

### 3.3 Single‐cell RNA sequencing

**FIGURE 5:**
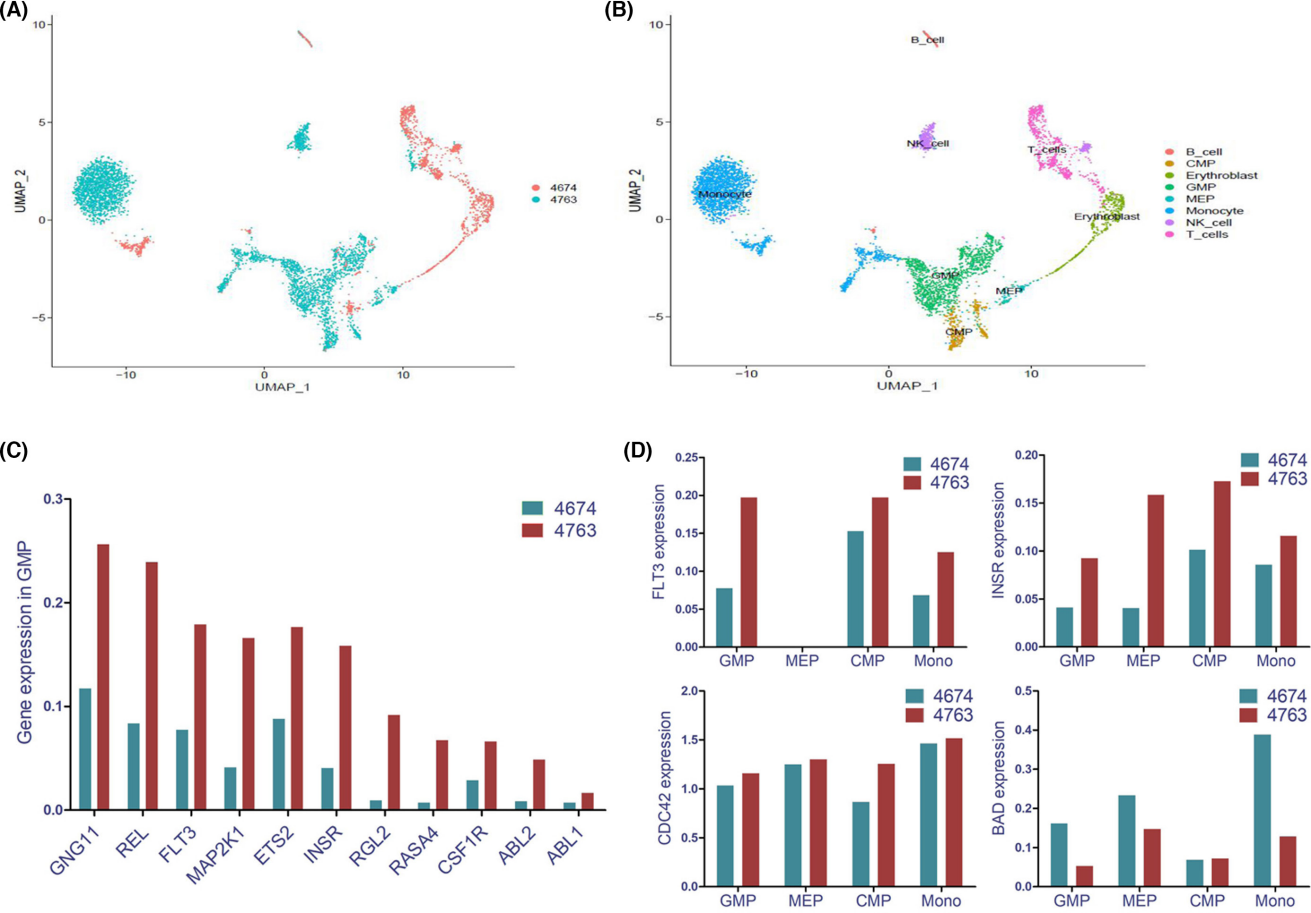
Single‐cell RNA transcription group sequencing revealed the linear association of gene mutations with the activation of targeted signalling. (A) Patient presented with several abnormal cell groups (blue) after disease progression (orange, 4674 before leukaemia transformation and blue, 4763 after leukaemia transformation). (B) Cluster analysis suggested that abnormal GMP, CMP, MEP and monocytes increased in the AML stage. Cells are integrated and shown in two samples. Cell types are recognized and shown in different colours. (C) Integrated analysis indicated that several *RAS* signalling‐related genes were highly expressed in GMP after disease progression. (D) RAS signalling‐related genes, such as *FLT3*, *INSR*, and *CDC42*, were also highly expressed in MEP, CMP, and Mono groups. However, the apoptosis‐related gene *BAD* is down‐regulated in GMP, MEP, and CMP.

As mentioned earlier, abnormal cell signalling induced by gene mutation, such as *RAS* genes or *PTPN11*, may be critical for the transformation of MDS into AML. However, it is still unclear whether a *RAS* mutation is a requirement to activate *RAS* signalling pathways. To explore this question, we used single‐cell RNA transcription sequencing to study the association between *RAS* mutation and *RAS* signalling in UPN4674 and UPN4763 (before and after disease progression). *NRAS* and *PTPN11* mutations occurring during the AML stage are core genes in *RAS* signalling. As shown in Figure 5A, B, one patient presented several abnormal cell types (orange, UPN4674; blue, UPN4763 in Figure 5A). Gene classification analysis showed that aberrant granular‐mononuclear progenitors (GMP), common myeloid progenitor (CMP), megakaryocyte‐erythroid progenitor (MEP), and monocytes (Mono) were present during the AML stage (Figure 5B). We focused on differences in the GMP population during *RAS* signalling. Integrated analysis based on single‐cell sequencing indicated that several *RAS* signalling‐related genes are expressed at high levels in GMP after disease progression (Figure 5C). These genes have been reported to participate in the activation of RAS signalling.^18^ Similarly, RAS signalling‐related genes such as *FLT3*, *INSR*, and *CDC42* are also expressed at high levels in MEP, CMP, and Mono groups after disease progression (Figure 5D). These genes are closely associated with cell proliferation. Interestingly, apoptosis‐related gene *BAD* is down‐regulated in GMP, MEP, CMP, and Mono groups after disease progression. These data suggest that TRMs gene mutations induce the redistribution of clonal cells, which leads to an increased number of morphological blasts and monocytes via the activation of cell signalling, and further leads to AML transformation.

## 4 DISCUSSION

The pathogenesis of sAML differs from that of de novo AML in several respects, including clinical progression and prognosis.^19^ Despite poor response to contemporary therapies, including stem‐cell transplantation, MDS‐derived AML exhibits a relatively long pre‐transformation duration and occurs only in about one‐third of all patients with MDS.^7^ If this transformation requires triggering factors (defined as TRMs), and these factors are robust biomarkers of progression, it may be possible to develop targeted therapy.

In this study, we sequenced 72 paired bone marrow DNA samples, first at MDS diagnosis and then immediately after AML transformation. Sixty‐four out of the 72 cases (88.9%) were positive for TRMs, that is, events involving active signalling, transcription factors, or tumour suppressors checked by targeted sequencing. Although the VAF value of TRMs showed no obvious increase after AML transformation in a number of patients (such as in patients 4, 15, 26, 31, 36, 43, 46, 47, 50, 54, 55, 60, 64), we consider that the longer existence of these TRMs genes would also promote progression. In part 4 of the targeted sequencing results section, we pointed out that TRM gene changes were sometimes ahead of the phenotype.

Sequencing of 39 target genes could not detect any TRMs in eight out of 72 cases. Of these, paired WES was performed for three cases for which sufficient DNA could be extracted from BM samples. Each of these patients carried at least one TRM (out of the 39 target genes) involving myeloid transcription or tumour suppressor genes (Figure 4). No patient with MDS in this assay who developed sAML did so only by acquiring mutations involved in epigenetic modulation, RNA splicing, or cohesins. This finding suggests that the existence or emergence of certain last event‐like mutations may be considered a prerequisite for MDS to transform into sAML rather than just occasional or accompanying events.^9^, ^10^

Of the 67 patients with TRMs detected, the TRMs appeared to emerge at the point of AML transformation in 43 patients (64.2%; including 40 by targeted sequencing and three by WES), and the remaining 24 patients harboured putative last mutations at the time of MDS diagnosis. Therefore, it is necessary to examine which the original is. Is it the existence or emergence of TRMs? Or the AML transformation itself? We considered that the pre‐existence or emergence of TRMs is the reason for AML transformation. First, 24 of the 67 (35.8%) cases harboured TRMs at MDS diagnosis. The transformation duration was moderately shorter in these cases than in those where the TRMs were only identified at the transformation point (mean eight vs. 11 months). Second, due to the relatively longer corresponding time between the diagnosis and AML transformation for paired sequencing, some important information may have been missed. When additional time points were added, supportive data were obtained from some patients (UPN1243, 3288, and 4390 in Figure 2). When TRMs emerged in these patients, their disease was still at the low‐risk or pre‐AML stage but transformed to AML quickly. Finally, for one patient (UPN 4674), immediately after the occurrence of the AML phenotype, accompanied by *NRAS* and *PTPN11* mutations, single‐cell RNA sequencing showed both active RNA transcription and activation of the *RAS/PTPN11* signalling pathway.^20^, ^21^ Given that the gene mutations occurred prior to RNA/protein transcription/translation, pathway activation, and, ultimately, phenotype alteration, these results support our findings that TRMs and activating *RAS* signalling can precede phenotypic transformation to AML. In a review, Dr. Menssen et al. elaborated that mutations in transcription factors and signalling pathway activation genes emerged commonly at MDS progression to sAML, similar to our findings.^22^

Most of the TRM clones showed linear evolution from the founding clones (29 cases, compared to five cases with linear evolution in a pre‐existing subclone; and six cases by clone sweeping) (Figure 3). This is somewhat different from previous reports,^23^, ^24^ which may be because a subset of the patients did not represent the clonal progressing process, such as the 24 cases whose TRMs pre‐existed at MDS diagnosis. In addition, we detected common pre‐existing mutations involving *TET2*, *RUNX1*, *ASXL1*, *DNMT3A*, *U2AF1*, *STAG2*, *ROBO1*, and *SF3B1* (Table 2), indicating that these mutations represent precursor mutations for these sAML cases. We reported a poor prognosis for patients with MDS that had *ROBO* mutations.^25^ This may be due to the non signalling/transcriptor/tumour suppressor function of *ROBO*, which may assist TRMs to finish AML transformation. As for *ASXL1*/*BCOR*/*TET2* mutations, they emerged as common partners of TRMs (Table 2), possibly playing a role in the transformation process. More research should be conducted to explore the relationship between *BCOR* mutations (usually occur in patients with normal chromosomes) and sAML,^26^ especially in patients with sAML and *RAS* mutations as TRMs (Table 2). Some mutated genes, such as *RUNX1* (highly prevalent among patients with chromosome seven abnormalities),^27^ could be responsible for the MDS phenotype or driving AML progression. These dynamic genetic features contribute to the complexity and heterogeneity of this disease and may become key to understanding the occurrence of sAML.

Together with *TP53*, mutations in *NRAS*/*KRAS*, *CEBPA*, *FLT3*, *CBL*, *PTPN11* and *RUNX1* (a total of 7 genes) accounting for almost 90% of patients for whom TRMs were defined by targeted sequencing. *KIT* mutation was not detected as a TRM in our group of cases, and *NPM1* was only detected in one case. The latter could be attributed to the favourable responses of the MDS patients who harbour NPM1 mutations toward HMAs (decitabine), thus blocking sAML transformation.^28^ We reported *CEBPA* as one of the most common TRMs (accounting for nearly one‐sixth of all TRMs) and expanded the candidate genes for possible targeted treatment. In addition, the mutation pattern of *FLT3* in this assay was less involved in *FLT3‐ITD* (only in one of the 8 cases; often observed in de novo AML). Therefore, it appears that point‐mutations‐aimed target therapy would be beneficial for patients with this kind of TRM. Given the rapid development of mutation‐specific targeted therapy in recent years, we hope to pharmacologically block AML transformation in patients with MDS at high‐transformation risk with regular monitoring for TRMs and the administration of effective corresponding targeted therapy.

There are several limitations to this study. This was not a prospective study. Therefore, some data from AML‐transformed cases could not be collected. Moreover, not all progression‐related genes were included in the 39 targets in this assay, which may have resulted in the exclusion of some useful information. A more exact design is needed to fully explore the sAML transformation process.

In summary, somatic mutations in signalling pathway activation, transcription factors, or tumour suppressor genes appear to be a precondition for AML transformation in MDS. The high propensity to acquire TRM is worthy of further research so that targeted novel therapies can be developed.

## AUTHOR CONTRIBUTIONS

**Feng Xu:** Data curation (equal); formal analysis (equal); validation (equal). **Lin‐Yun Wu:** Data curation (equal); formal analysis (equal); validation (equal). **Juan Guo:** Methodology (lead). **Qi He:** Investigation (lead). **Zheng Zhang:** Formal analysis (supporting); methodology (supporting). **Xiao Li:** Conceptualization (lead); funding acquisition (lead); supervision (lead); writing – review and editing (lead).

## FUNDING INFORMATION

This study was supported by the National Natural Science Foundation of China (grant nos. 81770120 and 81770122).

## CONFLICT OF INTEREST

The authors do not have any conflict of interest to declare.

## PATIENT CONSENT

Clinical and hematologic data from patients were recorded following informed consent in accordance with the Declaration of Helsinki.

## PERMISSION TO REPRODUCE MATERIAL FROM OTHER SOURCES

Not applicable.

## CLINICAL TRIAL REGISTRATION

Not applicable.

## Data Availability

The data can be available by contacting the corresponding author.
